
JOURNAL INFORMATION
==============================
NLM Title Abbreviation: J Gerontol A Biol Sci Med Sci
Journal ID: J Gerontol A Biol Sci Med Sci
NLM Journal ID: 9502837
Journal ID: gerona

The Journals of Gerontology Series A: Biological Sciences and Medical Sciences

ISSN: 1079-5006
EISSN: 1758-535X

ARTICLE INFORMATION
==============================
PMCID: PMC8344705
PMID: 34171089
DOI: 10.1093/gerona/glab184
Article ID: glab184
Article version: 1
Subjects: Letter to the Editor, AcademicSubjects/MED00280, AcademicSubjects/SCI00960

APOE e4 genotypes increase risk of delirium during COVID-19 related hospitalizations: evidence from a large United Kingdom cohort

https://orcid.org/0000-0003-4452-2380 Kuo Chia-Ling PhD 1 2
https://orcid.org/0000-0002-3332-8454 Pilling Luke C PhD 3
https://orcid.org/0000-0003-4919-9068 Atkins Janice L PhD 3
Fortinsky Richard H PhD 2
Kuchel George A MD 2
Melzer David MBBCh, PhD 3
1 Connecticut Convergence Institute for Translation in Regenerative Engineering, University of Connecticut Health , Farmington
2 Center on Aging, University of Connecticut Health , Farmington
3 Epidemiology and Public Health Group, University of Exeter Medical School , UK
correspondence: Chia-Ling Kuo 195 Farmington Avenue, Suite 2080, Farmington, CT 06032-6022 kuo@uchc.edu
Electronic publication date: 2021 Jun 25
Electronic Location ID: glab184
Received 2021 May 10
Copyright: © The Author(s) 2021. Published by Oxford University Press on behalf of The Gerontological Society of America.
Copyright year: 2021
License: This is an Open Access article distributed under the terms of the Creative Commons Attribution License (http://creativecommons.org/licenses/by/4.0/), which permits unrestricted reuse, distribution, and reproduction in any medium, provided the original work is properly cited.
License URL: https://creativecommons.org/licenses/by/4.0/

article-lifecycle: PAP
edited-state: accepted-manuscript

==============================
### *APOE* e4 genotypes increase risk of delirium during COVID-19 related hospitalizations: evidence from a large United Kingdom cohort

Chia-Ling Kuo, PhD[1,2]*, Luke C. Pilling, PhD[3], Janice L. Atkins, PhD[3], Richard H. Fortinsky, PhD[2], George A. Kuchel, MD[2], David Melzer, MBBCh, PhD[3]

[1]Connecticut Convergence Institute for Translation in Regenerative Engineering, University of Connecticut Health, Farmington.

[2]Center on Aging, University of Connecticut Health, Farmington.

[3]Epidemiology and Public Health Group, University of Exeter Medical School, UK.

*correspondence:

Chia-Ling Kuo

195 Farmington Avenue, Suite 2080, Farmington, CT 06032-6022

kuo@uchc.edu

Accepted Manuscript

© The Author(s) 2021. Published by Oxford University Press on behalf of The Gerontological Society of America.
This is an Open Access article distributed under the terms of the Creative Commons Attribution License (http://creativecommons.org/licenses/by/4.0/), which permits unrestricted reuse, distribution, and reproduction in any medium, provided the original work is properly cited.

Delirium is an acute confusional state, and is reported to be particularly frequent and severe in older adults with severe COVID-19 infection [1]. Little is currently known of the mechanisms linking COVID-19 to delirium. Given that the *APOE* e4 allele has been associated with delirium [2] and risk of having severe COVID-19 [3,4] in the general population, we hypothesized that the *APOE* e4 allele may be associated with increased risk of delirium during COVID-19 related hospitalizations.

To test the hypothesis, we used data from European-ancestry participants of the UK Biobank, recruited at England assessment centers in 2006-2010, with ages between 40 and 70 (n=398,034). The *APOE* genotype was determined based on the genotypes at the single nucleotide polymorphisms, rs429358 and rs7412, on chromosome 19. Participants with any hospital admission records after the first COVID-19 positive result were included. Delirium cases (ICD-10 codes: F05.0; F05.1; F05.8; F05.9) were restricted to those in the Acute Phase (AP delirium; within 30 days after test-confirmed COVID-19) [5]. Those diagnosed after 30 days (long COVID) were too few to analyze. Fine-and-Gray competing risk models accounting for the competing risk of mortality were used, adjusting for age, sex, and the top five genetic principal components from the European-ancestry participants.

375,173 participants of European descent, visiting England assessment centers at recruitment, were at risk of COVID-19 infection, excluding those who died before the first test date in the data (March 16, 2020, n=22,861). 8,725 were tested positive between March 16 and December 31, 2020, of whom those with e3e4 (n=2,074), e4e4 (n=251), or e3e3 (n=5,098) genotype were further considered. Of the sub-sample above (n=7,423), 51.6% were female and their mean age at the start of the pandemic (set as March 16, 2020) was 65.8 years (SD=8.7). Additionally, 1,363 (18.4%) were hospitalized after the first COVID-19 positive result, of whom 958 had COVID-19 diagnosis codes (ICD-10 codes: U071,

Accepted Manuscript

U072) in their hospital admission records. 546 died after being tested positive, of whom, 414 with causes of death including COVID-19 (mortality data updated to March 2021). Median follow-up time from first COVID-19 positive result to death or last follow-up of hospital admission data (December 31, 2020) was 56 days (range=0-290 days).

In the e4-delirium association analysis, 1,363 participants were hospitalized after test-confirmed COVID-19, of whom 358 were e3e4s (n=49 delirium cases, 43 AP), 64 were e4e4s (n=14, 13 AP), and 941 were e3e3s (n=66, 52 AP). e3e4 and e4e4 genotypes accounted for 47% (56/118) of AP delirium cases from all hospitalized after test-confirmed COVID-19 (n=1,575), and 52% (56/108) of AP delirium cases from those restricted to e3e4s, e4e4s, and e3e3s only. Both e3e4s ($HR_{e3e4}$=2.29, 95% CI: 1.54-3.41, p=$4.80\times10^{-5}$) and e4e4s ($HR_{e4e4}$=4.94, 95% CI: 2.69-9.09, p=$2.80\times10^{-7}$) were at higher risk of AP delirium compared to e3e3s. For sensitivity analysis, we used only those with a COVID diagnosis during hospitalization. We also analyzed hospitalized patients excluding those with pre-existing dementia before the first COVID positive date, and excluding one in third-degree or closer pairs, and the results were consistent in all analyses (Table 1).

For exploratory purposes, the associations between e4 genotypes and dementia are well established [6], and could possibly hold in patients with test-confirmed COVID-19. Currently, most of new dementia cases after test-confirmed COVID-19 were recorded in the AP (39 of 55 in e3e4, e4e4, or e3e3 genotypes). While we found that e4 genotypes were associated with increased risk of AP dementia ($HR_{e3e4}$=2.24, 95% CI: 1.13-4.44, p=0.02; $HR_{e4e4}$=7.86, 95% CI: 3.06-20.18, p=$1.8\times10^{-5}$), some of these patients likely developed dementia before COVID-19 infection, which was not captured in the hospital admission records until COVID-19 related hospitalizations. When more post COVID dementia cases are diagnosed, using additional primary care data would help exclude those with pre-existing dementia and a wash-out period may be set to confirm incident cases.

Accepted Manuscript

To conclude, we previously showed that *APOE* e4 genotypes are associated with increased COVID-19 severity [3,4]. In this letter, we add that delirium is enriched in e4 genotypes after test-confirmed COVID-19. Inevitably, our study has limitations: included samples restricted to white, hospitalized COVID-19 patients; no proper COVID-free controls to compare with for e4-delirium associations; limited data at the pandemic to characterize the samples and to test robustness of the associations. Our findings provide early evidence and need to be confirmed by independent replication in larger samples. Future investigations are needed to understand the underlying mechanisms and to study those not severely infected or hospitalized for long-term health impact from COVID-19 infection.

### Funding

C.L.K., L.C.P., G.A.K., and D.M. are supported in part by an R21 COVID-19 supplement grant (R21AG060018-02S1), funded by National Institute on Aging, National Institutes of Health, USA.

Accepted Manuscript

### References

1. Kennedy M, Helfand BKI, Gou RY, Gartaganis SL, Webb M, Moccia JM, Bruursema SN, Dokic B, McCulloch B, Ring H, Margolin JD, Zhang E, Anderson R, et al. Delirium in Older Patients with COVID-19 Presenting to the Emergency Department. JAMA Netw Open. 2020; 3: e2029540.

2. Pilling LC, Jones LC, Masoli JAH, Delgado J, Atkins JL, Bowden J, Fortinsky RH, Kuchel GA, Melzer D. Low Vitamin D Levels and Risk of Incident Delirium in 351,000 Older UK Biobank Participants. J Am Geriatr Soc. 2021; 69: 365–72.

3. Kuo CL, Pilling LC, Atkins JL, Masoli JAH, Delgado J, Kuchel GA, Melzer D, Newman AB. ApoE e4e4 Genotype and Mortality with COVID-19 in UK Biobank. Journals Gerontol - Ser A Biol Sci Med Sci [Internet]. 2020; 75: 1801–3. Available from: http://10.0.4.69/gerona/glaa169

4. Kuo CL, Pilling LC, Atkins JL, Masoli JAH, Delgado J, Kuchel GA, Melzer D. APOE e4 genotype predicts severe COVID-19 in the UK biobank community cohort. Journals Gerontol - Ser A Biol Sci Med Sci [Internet]. 2020; 75: 2231–2. Available from: https://doi.org/10.1093/gerona/glaa131

5. Gorna R, MacDermott N, Rayner C, O'Hara M, Evans S, Agyen L, Nutland W, Rogers N, Hastie C. Long COVID guidelines need to reflect lived experience. Lancet (London, England). 2021; 397: 455–7.

6. Pereira JVB, Aung Thein MZ, Nitchingham A, Caplan GA. Delirium in older adults is associated with development of new dementia: a systematic review and meta-analysis. Int J Geriatr Psychiatry. England; 2021; 36: 993–1003.

Accepted Manuscript

**Table 1 Numbers of subjects with delirium by *APOE* genotype and associations between *APOE* e4 genotypes and delirium in the acute phase (AP) after tested-confirmed COVID-19**

| **AP Delirium** | **#e3e3s (#AP delirium, %)** | **#e3e4s (#AP delirium, %)** | **#e4e4s (#AP delirium, %)** | **$HR_{e3e4}$ (95% CI)** | **$HR_{e4e4}$ (95% CI)** |
|---|---|---|---|---|---|
| positive + hospitalized, excluding non-AP delirium (n=1,342) | 927 (52, 5.6%) | 352 (43, 12.2%) | 63 (13, 20.6%) | 2.29 (1.54-3.41) $p=4.80\times10^{-5}$ | 4.94 (2.69-9.09) $p=2.80\times10^{-7}$ |
| positive + hospitalized + in-patient COVID-19 diagnosis, excluding non-AP delirium (n=948) | 655 (49, 7.5%) | 247 (42, 17.0%) | 46 (13, 28.3%) | 2.43 (1.62-3.64) $p=1.80\times10^{-5}$ | 5.13 (2.83-9.30) $p=7.0\times10^{-8}$ |
| positive + hospitalized, excluding pre-existing dementia and non-AP delirium (n=1,236) | 877 (43, 4.9%) | 309 (29, 9.4%) | 50 (7, 14%) | 2.07 (1.29-3.30) p=0.0024 | 3.74 (1.64-8.52) p=0.0017 |
| positive + hospitalized, excluding one in third-degree or closer pairs and non-AP delirium (n=1,094) | 764 (47, 6.2%) | 280 (29, 10.4%) | 50 (13, 26.0%) | 1.82 (1.15-2.88) p=0.011 | 6.47 (3.56-11.75) $p=9.0\times10^{-10}$ |

Hazard ratio comparing e3e4s to e3e3s ($HR_{e3e4}$) and that comparing e4e4s to e3e3s ($HR_{e4e4}$) from Fine-and-Gray competing risk models accounting for the competing risk of mortality, adjusting for age, sex, and the top five genetic principal components from the European-ancestry participants in the UK Biobank

Accepted Manuscript
